# Assessment of a psychoeducational group program for enhancing resilience and reducing burnout in primary healthcare workers in Catalonia: A pre-post study

**DOI:** 10.1016/j.aprim.2026.103483

**Published:** 2026-03-25

**Authors:** Enric Aragonès, Sara Rodoreda, Meritxell Guitart, Eva Garcia, Anna Berenguera, Francisco Martín-Luján, Concepció Rambla, Guillem Aragonès, Antoni Calvo, Ariadna Mas, Meritxell Pallejà, Josep Basora

**Affiliations:** a Primary Care Area Camp de Tarragona, Institut Català de la Salut, Tarragona, Spain; b Institut Universitari d’Investigació en Atenció Primària Jordi Gol (IDIAPJGol), Barcelona, Spain; c Primary Care Division, Institut Català de la Salut, Barcelona, Spain; d Primary Care Emotional and Community Wellbeing Program, Department of Health, Government of Catalonia, Barcelona, Spain; e Primary Care Centre Sant Vicenç de Castellet, Institut Català de la Salut, Barcelona, Spain; f Occupational Risk Prevention Unit, Institut Català de la Salut, Barcelona, Spain; g Universitat Rovira i Virgili University, School of Medicine and Health Sciences – Department of Medicine and Surgery, Reus, Spain; h Intitut d’Investigació Sanitària Pere Virgili (IISPV), Transversal Lifestyle and Disease Prevention Program, Tarragona, Spain; i Galatea Foundation, Barcelona, Spain; j General Directorate of Health Planning and Research, Department of Health, Government of Catalonia, Barcelona, Spain

**Keywords:** Mental health, Health workers, Primary healthcare, Burnout, Psychological resilience, Psychological support, Salud mental, Profesionales de la salud, Atención primaria, Desgaste profesional (burnout), Resiliencia psicológica, Apoyo psicológico

## Abstract

**Objective:**

To evaluate the feasibility and potential effectiveness of a structured psychoeducational group program aimed at enhancing resilience and reducing burnout among primary healthcare workers.

**Design:**

A single-arm pre–post implementation study conducted between September 2022 and February 2024. Trial registration at ClinicalTrials.gov: NCT05720429.

**Site:**

81 primary care centers of the Catalan Health Institute.

**Participants:**

Primary care professionals from all occupational profiles were eligible.

**Interventions:**

A 11-session program, delivered by community psychologists, combined psychoeducational content, interactive activities, and relaxation techniques.

**Main measurements:**

Outcomes were measured before and immediately after the intervention using the Connor–Davidson Resilience Scale and the burnout subscale of the Professional Quality of Life Scale. Analyses included effect sizes and multivariate models to identify predictors of change.

**Results:**

Of 1419 baseline participants, 387 (87.1% women; median age 47 years) completed both assessments. Resilience increased significantly post-intervention (*p* = 0.001; effect size = 0.21), with larger gains in men and younger participants. Burnout decreased significantly (*p* = 0.001; effect size = 0.21), particularly among physicians. Higher baseline secondary traumatic stress predicted greater burnout reduction. Program overall assessment, feasibility and satisfaction were high.

**Conclusions:**

This psychoeducational group intervention was feasible, well-received, and associated with modest but significant improvements in resilience and burnout. Targeted benefits were observed for specific subgroups, suggesting value in tailoring content to professional role and baseline emotional burden.

## Abstract (es)

**Objetivo:**

Evaluar la viabilidad y la efectividad de un programa grupal psicoeducativo para fortalecer la resiliencia y reducir el burnout entre los profesionales de atención primaria.

**Diseño:**

Estudio de implementación con un solo grupo y mediciones pre y post-intervención. Registro en ClinicalTrials.gov: NCT05720429.

**Emplazamiento:**

81 centros de atención primaria del Institut Català de la Salut.

**Participantes:**

Profesionales de atención primaria de diversos perfiles laborales.

**Intervenciones:**

Programa de 11 sesiones impartido por psicólogos comunitarios, combinando contenidos psicoeducativos, dinámicas participativas y técnicas de relajación.

**Mediciones principales:**

Las variables de resultado se evaluaron antes y después de la intervención mediante la *Connor–Davidson Resilience Scale* y la subescala de burnout de la *Professional Quality of Life Scale*. Se calcularon tamaños del efecto y se aplicaron modelos multivariantes para identificar predictores del cambio.

**Resultados:**

De los 1.419 participantes iniciales, 387 (87,1% mujeres; edad mediana 47 años) completaron ambas evaluaciones. La resiliencia aumentó significativamente tras la intervención (p = 0,001; tamaño del efecto = 0,21), especialmente en hombres y participantes más jóvenes. El burnout se redujo significativamente (p = 0,001; tamaño del efecto = 0,21), especialmente en médicos. El nivel basal de estrés traumático secundario se asoció con una mayor reducción del burnout. La viabilidad, la aceptación y la valoración global del programa fueron elevadas.

**Conclusiones:**

El programa grupal psicoeducativo demostró ser viable, bien valorado y asociado con mejoras modestas en la resiliencia y el burnout. Los resultados sugieren beneficios diferenciales según perfil profesional y carga emocional inicial, lo que refuerza la conveniencia de adaptar el contenido a las características de los participantes.

## Introduction

The mental health of primary healthcare workers has gained attention, particularly after the extraordinary stress of the COVID-19 pandemic. Rapid system changes during the crisis placed unprecedented demands on professionals, increasing workload, distress, and burnout risk.^1^, ^2^ Although the acute phase has passed, its longer-term impact has underscored pre-existing stressors in healthcare. Primary care staff still report high work-related stress that, if unaddressed, may contribute to mental disorders, lower job satisfaction, and burnout.^3^, ^4^ These pressures are often compounded by growing patient complexity, administrative demands, and staffing constraints. Moreover, primary care's gatekeeping role and longitudinal patient relationships can intensify emotional labor, making scalable, preventive interventions relevant for maintaining service quality in resource-limited health systems.

Burnout is characterized by emotional exhaustion, depersonalization, and reduced personal accomplishment.^5^ In primary care it affects not only individual well-being but also performance and patient safety; it has been linked to absenteeism, reduced productivity, and higher risk of medical errors.^6^, ^7^ Preventing and mitigating burnout is therefore important for workforce sustainability.

Resilience, the capacity to adapt positively to stress and adversity,^8^ supports effective coping and functioning in high-pressure environments. As a dynamic process, it can be strengthened through targeted interventions^9^ and is considered protective against burnout.^10^

Accordingly, resilience-promoting programs have been developed in multiple formats (multimedia, face-to-face; individual or group). Group psychoeducational interventions, often combining cognitive-behavioral strategies, mindfulness, and peer support, may foster well-being and professional fulfillment by providing practical stress-management skills. However, evidence for effectiveness remains mixed, with heterogeneous designs, variable outcomes, and limited real-world implementation data, underscoring the need for further evaluation.^11^, ^12^

In Catalonia, the Catalan Health Institute implemented initiatives to enhance resilience and prevent burnout, including a structured psychoeducational group program for primary care professionals. This study assessed the program's feasibility and clinical impact on resilience and burnout and explored predictors of improvement.

## Methods

This single-arm implementation study used a pre-post design to assess intervention effects in real-world clinical settings. Data were collected from September 2022 to February 2024. The protocol was approved by the Jordi Gol i Gurina IDIAP Clinical Research Ethics Committee (Barcelona; 27/05/2022; 22/086-PCV). All participants provided informed consent. The study was registered at ClinicalTrials.gov (NCT05720429), and the protocol is described elsewhere.^13^

Setting and participants: The intervention targeted the primary care centers under the Catalan Institute of Health.^14^ All centers that implemented the program during the study period were included; no sampling strategy was used, consistent with real-world implementation.

Eligible participants included all professional profiles, such as nurses and auxiliary nurses, family doctors, pediatricians, dentists, dental hygienists, physiotherapists, nutritionists, social workers, and administrative staff participating in the psychoeducational groups. Participants voluntarily enrolled in the psychoeducational program at their respective centers and completed the evaluation questionnaires. No random or consecutive sampling was applied. Exclusion criteria included the presence of a severe mental disorder or ongoing litigation for work-related psychological disability. Analyses were restricted to participants who completed both pre- and post-intervention questionnaires. The distribution of participating primary care centers and healthcare workers across Catalan provinces is presented in Table 1.

**Table 1:** Geographic distribution of participating Primary Care Centers (PCC) and healthcare workers across Catalonia.

Province	Number of PCC	%	Healthcare workers (*n*)	% of total
Barcelona	51	63.0%	204	52.7%
Girona	10	12.3%	54	14.0%
Lleida	7	8.6%	42	10.9%
Tarragona	13	16.1%	87	22.5%

Total	*81*	*100%*	*387*	*100%*

The intervention was a group psychoeducational program developed within the Primary Care Emotional Well-being and Community Health Program, led by the Catalan Institute of Health and the Catalan Department of Health.^15^ It was designed by a working group of expert community psychologists and integrates evidence-based psychoeducational and psychological strategies to promote resilience and emotional well-being and prevent burnout.

Sessions were delivered by community psychologists based in primary care centers. The program comprised 11 in-person sessions (weekly or biweekly), lasting 45–60 min and held during work hours. Each session followed a structured format (brief theoretical input, interactive exercises, guided meditation), with minor local adaptations permitted. The intervention is intended for long-term integration into routine primary care services. Session content is summarized in Table 2, and full details are reported elsewhere.^13^

**Table 2:** Content and structure of the psychoeducational program.

Topic	Description
Emotional management	Recognizing and regulating one's own emotions.
Thought management	Inner dialog, irrational beliefs, and the contrast between rational and distorted thoughts.
Stress management	Understanding stress, its causes, coping strategies, and its impact on health.
Communication skills	Assertiveness, communication styles, empathy, and active listening.
Self-care	Definition, importance, and the different dimensions of self-care.
Individual and group self-esteem	Concepts of self-esteem, its significance, relationship with self-concept, and traits of low self-esteem.
Anxiety and panic coping – mindfulness	Introduction to anxiety and stress concepts, along with basic mindfulness practices.
Motivation activation	Theoretical foundations of motivation and strategies for self-motivation.
Problem solving	Definition, conflict management, and effective problem-solving techniques.
Positive psychology and emotional intelligence	Positive emotions, resilience, positive work attitudes, engagement, and the flow experience.
Emotional expression through art	Health benefits derived from artistic expression.

Each session follows a consistent format, including: (1) an initial content presentation by the facilitator; (2) interactive activities to reinforce learning; (3) guided relaxation exercises; and (4) reminders about available participant resources.

Measurements and data collection: Outcomes were measured at baseline and immediately post-intervention using standardized self-administered online questionnaires.

– Burnout: Assessed with the burnout subscale of the ProQOL-Health, a 30-item measure developed for healthcare workers.^16^, ^17^ Items are rated from 1 (“Never”) to 5 (“Always”). The ProQOL-Health includes five subscales: perceived support, compassion satisfaction, burnout, secondary traumatic stress, and moral distress.^16^ The burnout subscale comprises six items assessing hopelessness, exhaustion, and difficulty meeting job demands; scores were classified as low (6–12), average (13–23), or high (24–30). Other subscales were used to characterize baseline psychosocial profile but were not primary outcomes.
– Resilience: Measured with the Connor–Davidson Resilience Scale (CD-RISC 10).^18^, ^19^ This 10-item scale rates resilience-related attitudes and behaviors from 0 (“Not true at all”) to 4 (“True nearly all the time”); higher scores indicate greater resilience.

Burnout and resilience were selected as primary outcomes because they are clinically relevant and align closely with the program's intended effects. Other outcomes (e.g., depression, anxiety, stress, or additional ProQOL subscales) were not included in the main analyses to keep the focus on the constructs most directly targeted by the intervention and to limit multiple comparisons that could complicate interpretation. This prioritization enabled a clearer assessment of impact on the program's core objectives.

Baseline explanatory variables

– Sociodemographic and work-related characteristics: Age, gender, marital status, profession, and workplace.
– Health status: Item 1 of the SF-12 survey,^20^, ^21^ with five categories, from “excellent” to “poor”.
– Psychological state: Depression, Anxiety, and Stress Scales (DASS-21),^22^, ^23^ which comprises depression, anxiety, and stress subscales. Each subscale contains seven items rated from 0 (“did not apply to me at all”) to 3 (“applied to me very much/most of the time”).
– Mental health history: Past/current mental disorders, current psychopharmacological or psychotherapeutic treatments.
– Program adherence: Number of sessions attended.

The feasibility of the program was evaluated in two ways. First, program adherence was assessed by calculating the percentage of sessions attended by each participant. Second, perceived utility was measured at post-intervention using a four-item ad hoc questionnaire developed by the research team and the psychologists involved in the design of the program. The items were defined to capture key aspects of perceived usefulness and feasibility of applying the acquired knowledge and skills to personal, family, and professional life, in line with the program objectives. Responses were rated on a six-point Likert scale, from “completely agree” to “completely disagree.” Overall satisfaction with the program was assessed using a five-point scale, ranging from “excellent” to “very deficient.”

No formal psychometric validation or pilot testing was conducted, as the questionnaire was intended for descriptive assessment of feasibility and perceived utility and not used as a validated outcome measure.

### Statistical analysis

No a priori sample size calculation was performed, as this was a real-world implementation study including all participants who took part in the program during the study period.

Descriptive analyses were conducted using frequencies and percentages for categorical variables, and medians and interquartile ranges for quantitative variables, as none of the variables followed a normal distribution. Group differences were assessed using the chi-square test for categorical variables when the assumptions of the test were met, and the Wilcoxon rank-sum test for quantitative variables. Normality was evaluated with the Shapiro–Wilk test, applying a significance level of 0.01 as the cutoff, given the large sample size and to avoid over-detection of minor deviations from normality.

Changes between pre- and post-intervention were evaluated by estimating the effect sizes. Since all variables were non-normally distributed, effect sizes were calculated using the rank-biserial correlation. In addition, the Wilcoxon signed-rank test applied for paired variables, and corresponding *p*-values are reported. All results were presented for the total sample and stratified by groups of interest.

Multivariate analyses were conducted using linear regression models to identify independent predictors of change in each outcome score. Change was defined as the difference between post-intervention and pre-intervention scores. Candidate variables were selected a priori based on clinical relevance. Final model selection was guided by the lowest Akaike Information Criterion (AIC), but the choice of the final multivariate model was ultimately determined by clinical relevance.

All analyses were performed with the statistical package R (R Foundation for Statistical Computing, Vienna, Austria; version 4.3.2), and results were considered significant with a *p*-value < 0.05.

## Results

The pre-intervention questionnaire included 1419 records; 387 individuals provided both pre- and post-intervention data. Participants with pre-only data were comparable to completers, with small differences in marital status and the ProQOL “Compassion Satisfaction” subscale; no other baseline variables differed significantly (Table 3).

**Table 3:** Baseline sociodemographic, occupational, and health characteristics of the sample: comparison between participants with both pre-intervention and follow-up assessments, and those with pre-intervention only.

	Total sample	Participants without follow-up assessment	Participants with follow-up assessment	*p* value
*Total*	*N* = 1419	*N* = 1032	*N* = 387	
*Age (years); median (IQR)*	47.0 (37.0–54.0)	47.0 (37.0–55.0)	47.0 (39.0–54.0)	0.669

*Gender*				
Women	1231 (87.9%)	894 (88.3%)	337 (87.1%)	0.816
Men	164 (11.7%)	115 (11.4%)	49 (12.7%)	
Non-binary	5 (0.4%)	4 (0.4%)	1 (0.3%)	

*Marital status*				
Single, divorced/separated, or widowed	469 (33.5%)	340 (33.6%)	129 (33.3%)	0.026
Married or living as a couple	930 (66.5%)	672 (66.4%)	258 (66.7%)	

*Profession*				
Physician	332 (23.4%)	252 (24.4%)	80 (20.7%)	0.106
Nurse	503 (35.6%)	360 (35.1%)	143 (37.0%)	
Auxiliary nurse	99 (7.01%)	70 (6.82%)	29 (7.5%)	
Administrative staff	372 (26.3%)	275 (26.8%)	97 (25.1%)	
Other staff	107 (7.6%)	69 (6.7%)	38 (9.8%)	

*Self-rated overall health status*				
Excellent, very good or good	1192 (86.4%)	857 (88.9%)	327 (87.4%)	0.366
Fair or poor	187 (13.6%)	129 (13.0%)	58 (15.0%)	
Lifetime mental disorder	154 (11.5%)	107 (11.1%)	47 (12.6%)	0.510
Current mental disorder	122 (9.16%)	89 (9.26%)	33 (8.89%)	0.919
Current mental health treatment, of which:	116 (8.64%)	83 (8.57%)	33 (8.85%)	0.955
Psychopharmacological	102 (87.2%)	74 (88.1%)	28 (84.8%)	0.759
Psychological	65 (55.6%)	48 (57.1%)	17 (51.5%)	0.730

*Emotional status*				
DASS-21 depression	3.0 (1.0–6.0)	3.0 (1.0–6.0)	3.0 (1.0–5.0)	0.174
DASS-21 anxiety	3.0 (1.0–5.0)	3.0 (1.0–5.0)	3.0 (1.0–5.0)	0.305
DASS-21 stress	7.0 (5.0–8.0)	7.0 (5.0–8.0)	7.0 (5.0–8.0)	0.844

*ProQOL-Health subscales*				
Perceived support	24.0 (22.0–27.0)	24.0 (22.0–27.0)	24.0 (22.0–27.0)	0.803
Compassion satisfaction	23.0 (20.0–26.0)	24.0 (21.0–26.0)	23.0 (20.0–26.0)	0.025
Burnout	16.0 (13.0–19.0)	16.0 (13.0–20.0)	16.0 (13.0–19.0)	0.287
Secondary traumatic stress	14.0 (12.0–17.0)	14.0 (12.0–17.0)	14.0 (11.0–17.0)	0.129
Moral distress	15.0 (13.0–17.0)	15.0 (13.0–17.0)	15.0 (13.0–17.0)	0.855
Compassion fatigue	24.0 (22.0–27.0)	24.0 (22.0–27.0)	24.0 (22.0–27.0)	0.803
CD-RISC score	29.0 (25.0–33.0)	29.0 (25.0–33.0)	29.0 (25.0–33.0)	0.421

*Notes*: Data are presented as median and interquartile range (IQR) and numbers and percentages. *p*-Values were determined using Wilcoxon Rank Sum test for continuous variables and *χ*^2^ test for categorical variables. Connor–Davidson Resilience Scale (CD-RISC) measured resilience; DASS-21: Depression, Anxiety, and Stress Scales; ProQOL: Professional Quality of Life Scale. Numbers may not add up to the total sample size due to missing data for some variables.

All analyses were restricted to the 387 completers from 81 primary care centers. Median age was 47.0 years (IQR 39.0–54.0), and 337 (87.1%) were women. The most common professional profiles were nurses (35.6%), administrative staff (25.1%), and physicians (20.7%). The median ProQOL burnout score was 16.0 (IQR 13.0–19.0), indicating an average burnout level; 20 participants (5.5%) had high burnout. Median CD-RISC was 29.0 (IQR 25.0–33.0). Detailed characteristics are shown in Table 3.

Changes in resilience and burnout:

- Resilience

Overall resilience increased significantly after the intervention (*p* = 0.001; small effect size = 0.21). Gains were largest in men (*p* = 0.001; 0.58), and significant in participants aged 19–45 years (*p* < 0.001; 0.37) and in women (*p* = 0.031; 0.15).

By professional group, physicians experienced the greatest improvement (*p* < 0.001; 0.51), and administrative staff also improved (*p* = 0.036; 0.27); no relevant changes were observed in nurses/auxiliary nurses or other profiles. Resilience increased both among those attending all/most sessions (*p* = 0.027; 0.19) and those with partial attendance (*p* = 0.009; 0.28), with slightly greater gains in the latter (Table 4).

- Burnout

**Table 4:** Changes in resilience and burnout before and after the intervention, analyzed by gender, age, and professional profile.

		Resilience				Burnout			
	*N*	Pre-intervention	Post-intervention	Effect size	*p* value	Pre-intervention	Post-intervention	Effect size	*p* value
*Total*	370	29 [25–33]	29 [26–33]	0.21	0.001	16 [13–19]	16 [12–18.25]	0.21	0.001

*Sex*									
Women	323	29 [25–33]	29 [26–33]	0.15	0.031	16 [13–19]	16 [12–18.25]	0.22	0.003
Men	46	29 [24–33.75]	29 [26–33]	0.58	0.001	16 [12.25–19.75]	15 [12–18.25]	0.15	0.407

*Age groups*									
19–45 y/o	147	29 [25–33]	30 [27–34]	0.37	<0.001	15 [12–18.5]	15 [12–18]	0.23	0.043
≥45 y/o	223	29 [25–33]	29 [26–33]	0.10	0.208	16 [13–20]	16 [13–19]	0.21	0.013

*Profession*									
Physician	75	27 [22–30.5]	29 [26–31.5]	0.51	<0.001	19 [16–22]	18 [15–20]	0.42	0.003
Nurse/auxiliary nurse	164	28.5 [25–33]	29 [26–33]	0.03	0.786	16 [13–18]	16 [13–18]	0.16	0.117
Administrative staff	93	30 [26–35]	31 [27–35]	0.27	0.036	15 [12.5–17.5]	14 [11–18]	0.15	0.263
Other staff	38	30 [25.25–33.75]	31 [27.5–35.25]	0.32	0.112	14 [10–17]	12 [10–16.5]	0.11	0.626

*Adherence to intervention*									
All or almost all sessions attended	225	29 [25–34]	29 [26–33.5]	0.19	0.027	16 [13–19]	16 [12–18]	0.11	0.218
Some or few sessions attended	135	28 [25–32.5]	29 [26–33]	0.28	0.009	16 [14–20]	16 [13–19]	0.41	<0.001

*Notes*: Resilience was measured using the Connor–Davidson Resilience Scale (CD-RISC), and burnout was assessed using the burnout subscale of the Professional Quality of Life Scale (ProQOL Health). Data are reported as median and interquartile range (IQR). *p*-Values were calculated using the Wilcoxon signed-rank test, and effect sizes were estimated with rank-biserial correlation: values < 0.1 indicate a negligible effect, 0.1 a small effect, 0.3 a medium effect, and 0.5 a large effect. Numbers may not add up to the total sample size due to missing paired outcome data (*n* = 17), one non-binary participant excluded from sex-stratified analyses, and 10 cases with missing adherence data.

Burnout decreased significantly after the intervention (*p* = 0.001; small effect size = 0.21), with a more marked reduction among physicians (*p* = 0.003; medium effect size = 0.42). By adherence, burnout decreased in participants with partial attendance (*p* < 0.001; 0.41), whereas no significant change was observed among those with high adherence (*p* = 0.218) (Table 4).

- Predictors of change in resilience and burnout

Regression models identified significant predictors of change in both outcomes (Table 5). For resilience, younger age (*β* = −0.062; *p* = 0.005) and male gender (*β* = 1.763; *p* = 0.011) were associated with greater improvement; no other variables, including baseline psychological measures, were independently associated.

**Table 5:** Multivariate analysis identifying independent predictors of change in resilience and burnout scores following participation in a psychoeducational program for primary healthcare workers.

Resilience				Burnout			
	*β*	95% CI	*p* value		*β*	95% CI	*p* value
Sex (man)	1.76	0.41 to 3.11	**0.011**	Sex (man)	−0.576	−1.55 to 0.40	0.245
Age (years)	−0.06	−0.10 to −0.02	**0.005**	Marital status: not married	0.655	−0.02 to 1.35	0.058
Self-rated health status: fair or poor	0.97	−0.35 to 2.28	0.149				
Lifetime mental disorder	−0.74	−2.11 to 0.63	0.290	Lifetime mental disorder	0.587	−0.41 to 1.59	0.249
ProQOL-H: Compassion satisfaction	−0.10	−0.22 to 0.03	0.125	ProQOL-H: Secondary traumatic stress	−0.206	−0.30 to −0.11	**<0.001**
DASS: Depression	0.12	−0.02 to 0.26	0.096	DASS: Stress	0.084	−0.02 to 0.19	0.111

*Notes*: CD-RISC: Connor–Davidson Resilience Scale; ProQOL-H: Professional Quality of Life Scale, Healthcare workers version; DASS: Depression, Anxiety and Stress Scales.

For burnout, higher baseline secondary traumatic stress predicted a greater reduction (*β* = −0.206; *p* < 0.001), with no significant associations for other demographic, labor, or clinical variables.

- Feasibility, suitability, and participant satisfaction

**Figure 1:**
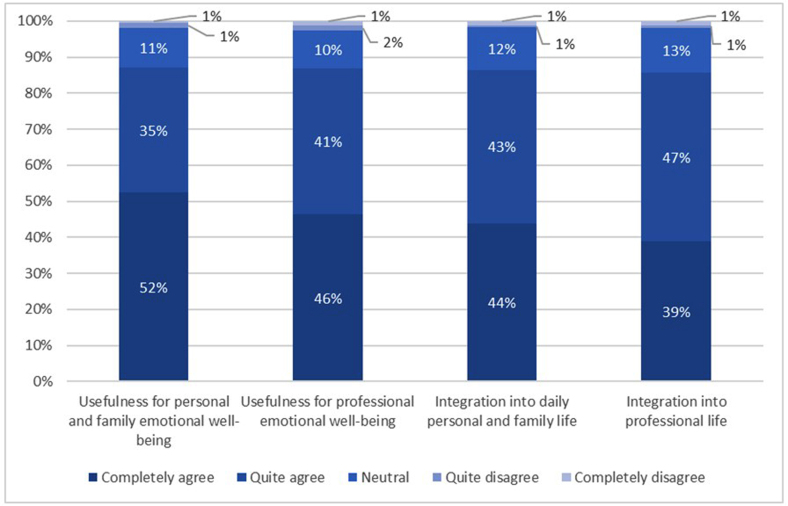
Participants’ perceptions of the usefulness and feasibility of integrating acquired learning, knowledge, and skills into personal, family, and professional life.

Of the 377 participants, 234 (62.1%) attended nearly all sessions, 99 (26.3%) attended ≥50%, and 33 (8.8%) attended <50%; attendance data were missing for 11 (2.9%). Most participants reported the program as useful for their personal and professional lives and considered the acquired skills feasible to apply (Fig. 1). Overall satisfaction was high: 48% rated the program as excellent, 43% as good, 7% as neutral, 2% as deficient, and none as very deficient.

## Discussion

This study evaluated the feasibility and potential effectiveness of a structured psychoeducational group program to enhance resilience and reduce burnout among primary healthcare professionals. Implemented at scale in routine practice, the program was well received, with high satisfaction and perceived usefulness. Resilience increased and burnout decreased significantly, although changes were modest and more marked in specific subgroups. Despite the limited magnitude, these results align with prior evidence that group interventions integrating cognitive-behavioral strategies, mindfulness, and peer support can produce small yet clinically meaningful improvements in healthcare workers’ psychological well-being, particularly in high-stress settings.^11^, ^12^, ^24^

Greater resilience gains among younger participants may reflect age-related differences in psychological flexibility and readiness to adopt new coping strategies, as reported in other interventions.^25^ Although men were a minority, they showed larger improvements; this finding should be interpreted cautiously and may relate to sex differences in baseline distress or coping styles.^26^

Stratified analyses showed that physicians experienced the largest reduction in burnout, consistent with evidence that, despite high emotional burden and risk of exhaustion, they may benefit from structured programs targeting emotional regulation and stress management.^27^, ^28^ In contrast, no significant changes were observed among nurses or administrative staff, diverging from systematic reviews reporting benefits in these groups.^29^ This may reflect contextual differences (role-specific stressors, organizational dynamics, engagement) and suggests that program content may need adaptation to better address distinct professional challenges.

Higher baseline secondary traumatic stress was associated with greater burnout reduction. Given its prevalence in emotionally demanding healthcare contexts, the program elements (guided reflection, peer discussion, emotional regulation) may be particularly relevant for these professionals and align with trauma-informed principles: safety, trust, validation, and peer support.^30^ Tailoring interventions to high emotional exposure may therefore enhance effectiveness, consistent with evidence supporting targeted approaches to improve healthcare workers’ well-being.^10^, ^11^

Unexpectedly, participants with partial attendance showed improvements comparable to those with high adherence, possibly due to self-selection and motivation. Some may have stopped once they perceived sufficient benefit, while others may have continued for reasons unrelated to perceived utility (e.g., institutional encouragement), yielding smaller relative change—patterns also reported in workplace well-being interventions.^12^ Overall, effects were modest, but targeted benefits may exist; tailoring by baseline vulnerability, professional role, or coping resources could increase relevance and impact.

### Strengths and limitations

Strengths include evaluation of a structured psychoeducational intervention delivered at scale in routine primary care, enhancing external validity and providing feasibility/acceptability data alongside effectiveness outcomes. The large, geographically diverse sample (81 primary care centers across Catalonia) closely reflects the target population, supporting generalizability.

Limitations include the absence of a control group, which precludes causal inference and leaves room for regression to the mean or natural fluctuations.^31^ However, because baseline burnout was in the average range and resilience was close to population norms,^32^ these influences are likely less pronounced. Second, no a priori sample size calculation was performed, as the study included all those who took part in the program during the study period; therefore, results should be interpreted as descriptive of this implementation context rather than as population-based estimates. Participation was voluntary and self-selected, potentially introducing selection bias,^33^ and in addition, neither primary care centers nor participants were selected through a formal sampling process, which limits the generalizability of the findings beyond similar implementation settings. Comparability between participants with and without follow-up suggests limited attrition bias. The lack of long-term follow-up prevents assessment of durability. Fifth, perceived utility was assessed using an ad hoc questionnaire developed for this study, without prior psychometric validation or pilot testing, which limits comparability with other studies and the precision of these feasibility-related findings. Finally, data collection over 17 months may have introduced time-varying external influences (e.g., organizational changes, workload) that could moderate impact.

### Practical implications and future research

This program appears feasible, acceptable, and potentially useful for promoting mental health in primary care. Integrating it into routine practice may support emotionally burdened professionals, particularly if content and delivery are adapted to different groups. Such interventions may also have a preventive role under acute or chronic stress, contributing to an institutional approach to workforce mental health.

Further randomized controlled trials with longer follow-up are needed to assess durability and functional outcomes (e.g., absenteeism, staff retention, perceived care quality). Future research should also examine tailored versions for underrepresented or less responsive groups and the feasibility and effectiveness of hybrid or digital formats to improve accessibility and implementation in resource-constrained settings.

**What is known about this topic**

• Primary care professionals face sustained work-related stress and are at risk of burnout, with implications for wellbeing and care quality.
• Group psychoeducational interventions may improve resilience and reduce burnout, but real-world effectiveness data are still limited.

**What this study adds**

• In a large real-world pre–post implementation study, a new program was feasible and well accepted, with high satisfaction and perceived usefulness.
• Resilience increased and burnout decreased significantly (modest effects), with larger gains in younger participants/men (resilience) and physicians, and greater burnout reductions in those with higher baseline secondary traumatic stress.

## Ethical considerations

The study was approved by the Research Ethics Committee of the IDIAP Jordi Gol (Barcelona, 27/05/2022; code 22/086-PCV), complying with the principles of the Declaration of Helsinki. Informed consent was obtained from the participants.

## Funding

The study is funded by the Department of Health (Government of Catalonia) by means of the Strategic Plan for Research and Innovation in Health (PERIS) 2022–2024 (SLT021/21/000028). EA is the beneficiary of a grant for intensifying research as senior researcher (IDIAPJGol; code 7Z21/007). The funding bodies have had no role in the design of the study or in the analysis and interpretation of data or its diffusion.

## Conflict of interest

The authors declare that they have no known competing financial interests or personal relationships that could have appeared to influence the work reported in this paper.
